# Differences in “minty” flavor compound and synthetic cooling agent presence in US-marketed menthol-mint e-liquids and devices between 2019–2023

**DOI:** 10.21203/rs.3.rs-8355233/v1

**Published:** 2025-12-16

**Authors:** Hanno C. Erythropel, Momoko Ishii, Deyri Garcia Torres, Mihnea-Andrei Petrescu, Penelope Venot-Medina, Remi A. Mellinghoff, Elena M. Bouldin, Paul T. Anastas, Barry G. Green, Stephanie S. O’Malley, Suchitra Krishnan-Sarin, Julie B. Zimmerman

**Affiliations:** Yale University; Yale University; Yale University; Lycée Pierre-Gilles-de-Gennes – ENCPB, Paris, France; Lycée Pierre-Gilles-de-Gennes – ENCPB, Paris, France; Yale University; Yale University; Yale University School of Medicine; The John B. Pierce Laboratory, New Haven, CT, USA; Yale University School of Medicine; Yale University School of Medicine; Yale University

**Keywords:** e-cigarette, e-liquid, ENDS, disposable, synthetic cooling agent, WS-23, WS-3, flavor, menthol, mint, nicotine, peppermint, spearmint, wintergreen

## Abstract

Menthol and mint flavors consistently rank among the most popular flavor categories in electronic nicotine delivery systems (ENDS). While menthol/mint is often as a combined flavor category, different distinct “minty” flavors arise from a range of chemically distinct compounds to produce peppermint aroma (menthol, menthone, menthyl acetate, eucalyptol), spearmint aroma (L-carvone), and wintergreen aroma (methyl salicylate). In addition, the synthetic cooling agents WS-23 and WS-3 have gained popularity since 2019. This study analytically characterized menthol/mint ENDS products marketed in the United States in 2019 and 2023 for these compounds, alongside ancillary nicotine analysis.

A total of 104 refill e-liquids purchased from online vendors in 2019 and 63 products purchased from online vendors in 2023 (including refill e-liquids, disposable devices, and pod-based products) were analyzed. In 2019 samples, menthol was prevalent (87/104 products; mean content 15.6 mg/g), whereas synthetic cooling agent use was rare (WS-23: 1/104; WS-3: 11/104). 2023 products showed decreased menthol concentrations (mean 8.3 mg/g), while synthetic coolant use became much more widespread (WS-23: 43/63; WS-3: 23/63). Synthetic coolant use prevalence was largely driven largely by disposable devices, all of which contained WS-23 at higher concentrations (mean 28.7 mg/g). Mint-, peppermint-, and spearmint-labeled products frequently contained overlapping flavorant profiles in both test samples, indicating limited reliability of label-based flavor categorization. Nicotine content closely matched labels for pod-based products but deviated substantially for disposables.

Overall, results indicate a marked shift from menthol toward synthetic cooling agents in U.S. marketed ENDS products between 2019 and 2023, particularly in disposable products. This underscores the importance of analytical verification of menthol/mint ENDS products, but also suggests that regulatory efforts to limit the use of menthol due to its known cooling effect should not be limited to the compounds menthol, but should encompass all compounds that activate the relevant receptor TRPM8.

## Introduction

The menthol/mint flavor category has regularly ranked among the top 3 flavor categories in surveys of those who use electronic nicotine delivery systems (ENDS) across age groups.^1–5^ Notably, a big uptake in youth preference for the menthol/mint category was observed in the US PATH study wave 5 (Dec. 2018 – Nov. 2019). This uptake coincided with the decision by the manufacturer of the then-popular Juul to stop selling mango, fruit, creme, and cucumber flavors in retail stores in Nov. 2018,^6^ while continuing to market both menthol and mint flavors until Nov. 2019,^7^ when mint flavor was also pulled voluntarily from the market likely in anticipation of updated enforcement priorities for cartridge based (refillable) ENDS published in April 2020.^8^

Following these changes in enforcement priorities, disposable devices, like Puff Bar, gained popularity due to a loophole that allowed their continued sale in all flavors because they were not cartridge based systems that could be refilled,^9^ which raised environmental concerns due to increased waste.^10^ Since then, more sophisticated devices like Elfbar have emerged on the market,^11^ which are sold in a multitude of flavors, including menthol and mint.^12^

While menthol and mint are often combined into one flavor category of menthol/mint in ENDS,^13^ there exist various “minty” flavors, including mint (no specification), peppermint, spearmint, and wintergreen.^14,15^ On the other hand, menthol is a single compound (but also the principal flavor compound in peppermint^16^), and a menthol-flavored e-liquid should thus only contain menthol as a single flavor compound. The main flavor compounds in peppermint (*Mentha × piperita*) are menthol, menthone, the menthol-based ester menthyl acetate, and 1,8-cineole (also called eucalyptol due to its high prevalence in eucalyptus leaves).^16^ Spearmint’s (*Mentha spicata*) predominant flavorant is L-carvone, which is often used in toothpaste and chewing gum,^16^ whereas Wintergreen’s characteristic flavor mostly stems from the flavorant methyl salicylate (usually *Gaultheria procumbens*).^17^

**Figure 1:**
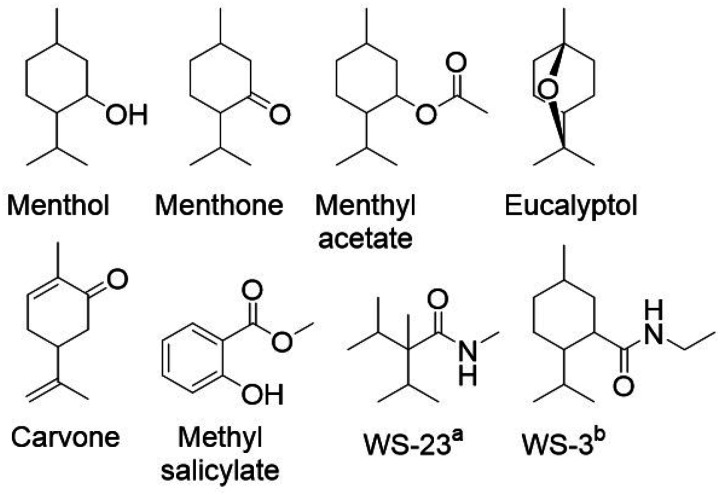
Chemical structures of “minty” flavor compounds and synthetic cooling agents tested for in this study. ^a^ WS-23: 2-isopropyl-N,2,3-trimethylbutyramide; CAS No. 51115–67-4. ^b^ WS-3: N-ethyl-p-menthane-3-carboxamide; CAS No. 39711–79-0.

Menthol has traditionally been used in various tobacco products and remains one of the few compounds exempt from flavor regulations on tobacco products in many jurisdictions around the world.^18^ Several physiological effects of mentholated cigarettes have been described,^19^ including that menthol is a TRPM8 cold receptor agonist which results in a cooling sensation and analgesic effect,^20,21^ which in turn can reduce the harshness of inhaled smoke toxicants and nicotine.^22,23^ Many recent studies have reported on the increasing prevalence of the synthetic coolants WS-23 and WS-3 starting with our report of European Juul containing WS-3,^24^ and WS-23 has since become a demonstrably ubiquitous additive in disposable devices in the US and Australia (see Fig. 1 for structures).^25–30^ Both WS-23 and WS-3 are named after the Wilkinson Sword company that pioneered their development since the 1960s for use in personal care products.^31^ Such synthetic cooling agents activate the same TRPM8 cold receptors as menthol but their lower vapor pressure results in lower volatility, rendering these compounds almost odorless.^31,32^

The goal of this study was to analytically determine the presence of the listed flavorants responsible for creating “minty” flavors (menthol, menthone, menthyl acetate, eucalyptol, L-carvone, methyl salicylate; see Fig. 1 for structures), and to quantify menthol and the synthetic cooling agents WS-23 and WS-3 in menthol/mint e-liquids marketed in the US before (2019) and after the rise of disposable devices (2023). Ancillary analysis included the determination of nicotine content.

## Methods

### Commercial Product Selection & Purchase

2019 products were identified using Google searches for “e-cigarette”, “e-juice”, “e-liquid”, and “vaping” in combination with “cool”, “ice”, “menthol”, “mint”, and “wintergreen”. Identified websites were then searched for relevant products which were purchased. A total of 104 products were purchased between March-December 2019 from a total of 27 websites (see Table S1 for details).

For 2023 e-liquid and disposable product selection, Google searches for “e-liquid”, “vape”, “disposable vape”, “ejuice”, and “e-cig” were carried out, and US websites marketing ENDS products were recorded. Semrush.com website traffic data was utilized to determine the number of unique US visitors to the collected websites in September 2023, and the 3 most popular websites were selected: elementvape.com (1.6 million unique visitors), mipod.com (371k), and eightvape.com (350k). These 3 websites were searched for “menthol” and “mint” products, website searches were sorted by popularity, and the top 10 disposable products were identified. When a brand in the top 10 products offered other relevant flavors, these were also purchased (e.g., Lost Mary-branded flavors ‘light snoow peppermint’ (sic), ‘Miami mint’, and ‘spearmint’). For refill e-liquids, which yielded < 10 results, all relevant products were purchased. A total of 32 disposable devices and 22 refill e-liquids were purchased in Nov. 2023 (see Table S2 for details). In addition, based on sales data during a 4-week period in December 2022 identifying top selling brands,^33^ relevant menthol/mint products by Vuse, Juul, NJOY (all pod products), and Breeze Smoke (disposable) were purchased (see Table S2 for details). Of note, the 5th identified top-selling brand, Elf Bar, was already identified in the website search.

Once received, products were sorted into 5 categories based on label information: Menthol, mint/peppermint, spearmint, wintergreen, and mixed/concept flavors. These categories were based and expanded upon the menthol/mint-section of the previously proposed e-liquid flavor wheel.^13^ Nicotine content and whether synthetic nicotine was mentioned on the label was also recorded.

### Chemicals & Materials

Commercial chemicals and standards were used as received. These included methanol (> 99.9% purity, Fisher Scientific, Waltham, MA), L-carvone (99%), 1,4-dioxane as internal standard (≥ 99%), eucalyptol (≥ 99%), menthone (≥ 97%), menthyl acetate (97%), methyl salicylate (≥ 99%), (−)-nicotine (≥ 99% all Sigma Aldrich, St. Louis, MO), N-ethyl-p-menthane-3-carboxamide (“WS-3”, ≥ 98%), 2-isopropyl-N,2,3-trimethylbutyramide (“WS-23”, ≥ 98%), (+)-menthol (≥ 98% all TCI America, Portland, OR).

### Sample Preparation, Characterization, and Compound quantification

Following the respective purchases, disposable and pod products were opened and liquid contents extracted and stored in glass vials, and refill e-liquids were used directly. Approximately 40–60mg of each e-liquid was weighed out (Sartorius Practum, Göttingen, Germany) and diluted in 1mL methanol containing 1mg/ml internal standard. Samples were characterized by gas chromatography-mass spectroscopy (GC-MS) to determine presence or absence of menthol, nicotine, WS-3, and WS-23, as well as the “minty” compounds menthone, menthyl acetate, eucalyptol, L-carvone, and methyl salicylate. Menthol, nicotine (both 2019 and 2023), WS-23, and WS-3 (2023 only) were then quantified by GC-flame ionization detection (FID) using established methods using single injections.^28^ WS-23 and WS-3 contents of those 2019 samples positively identified to contain synthetic coolants in 2019, were quantified in 2025 by GC-FID.

For GC-MS, 1μL was injected into a Clarus 580 GC – SQ8S MS outfitted with an Elite-5MS column (length 60m, id 0.25mm, 0.25 μm film; all PerkinElmer, Waltham, MA). Carrier gas: Helium. Split ratio: 10; Injector temperature: 300°C; Oven program: 40°C for 7min, ramp 10°C/min to 50°C and hold for 20min, ramp 10°C/min to 310°C and hold for 8min, ramp 10°C/min to 325°C and hold for 11.5min. MS settings: EI + mode with a mass range of m/z 30–620. For GC-FID, 1μL was injected into a GC-2010 (Shimadzu, Kyoto, Japan) outfitted with a J&W DB-5 column (same dimensions as GC-MS; Agilent, Santa Clara, CA). Carrier gas: Helium. Split ratio: 300; Injector temperature: 250°C; Oven program: 30°C for 7min, ramp 10°C/min to 50°C and hold for 20min, ramp 10°C/min to 310°C and hold for 12min. Detector temperature: 325°C.

Commercial standards were used to verify MS fragmentation patterns and GC-MS retention times to confirm compound identity, and to generate ≥ 5-point GC-FID calibration curves. GC-MS limits of detection (LOD) ranged from 2.5–20μm/mL; GC-FID LOD and limit of quantification (LOQ) were: 5μm/mL and 15μm/m, respectively. GC-FID Precision ranged from 0.73–0.80%RSD (7 injections; see Table S3 for details).

## Results

### 2019 products

All tested 2019 products were e-cigarette refill e-liquids. Menthol was found in 87/104 products with a mean concentration of 15.6mg/g, albeit with large variation as evidenced by a standard deviation of 31.0mg/g and ranging from 0.4–217.3mg/g (Table 1). Synthetic cooling agent prevalence was much lower, with 11/104 products containing WS-3, and only 1/104 products containing WS-23 (Table 2). At time of purchase, these were not quantified, but re-testing in 2025 showed a range of synthetic coolant presence of < LOQ – 10.3 mg/g. The other analyzed “minty” compounds menthone, menthyl acetate, and eucalyptol (peppermint constituents), L-carvone (characteristic of spearmint), and methyl salicylate (characteristic of wintergreen) were present in 25–68 of 104 products (Table 2).

Flavor categorization based on product labels was compared to the results of “minty” compound presence: 29/29 products marketed as menthol-flavored did contain menthol, however up to 17/29 products also contained other “minty” flavor compounds such as menthone (Table 2). 5 products also contained the spearmint flavorant L-carvone, and 3 products contained the wintergreen flavorant methyl salicylate. A majority of mint/peppermint-flavored products (> 15/23) contained characteristic peppermint flavorants, but 9/23 also contained L-carvone, which could suggest that the “mint” label could have also referred to spearmint or a mixture of peppermint and spearmint. Similarly, while 15/15 of spearmint-flavored contained L-carvone, 8–10 also contained compounds characteristic of peppermint, suggesting mixed peppermint/spearmint flavors. 9/10 wintergreen-flavored products did contain the characteristic flavorant methyl salicylate and only 1/10 contained menthol (but no methyl salicylate) and 1/10 contained both methyl salicylate and menthone (Tables 2 & S1). Finally, products with no distinct flavor descriptor in the mixed/concept category, such as ‘arctic rush’, ‘minty menthol’, or ‘wintermint’ showed a large variety of “minty” flavorant presence, ranging from 10/27 products containing L-carvone to 25/27 containing menthol. WS-23 was only detected in one product (‘winter ice’) in the mixed/concept category, while WS-3 was detected in a total of 11 products across all categories except wintergreen (Table 2 & S1).

### 2023 products

63 products procured in 2023 included refill e-liquids (22 products), disposable devices (35) and pod products (6). 60/63 products contained menthol with a mean concentration of 8.3mg/g (standard deviation: 4.1mg/g) and a range of 0.2–18.4mg/g; 43/63 contained WS-23 with a mean concentration of 25.0mg/g (13.2mg/g) and a range of 1.7–54.6mg/g; and 23/63 contained WS-3 with a mean concentration of 2.5mg/g (1.5mg/g; Table 1) and a range of 0.2–5.1mg/g. Notably, when breaking down menthol and synthetic coolant content by device type, stark differences become apparent: In refill e-liquids, mean menthol content was 8.8mg/g (4.5 mg/g) in 21/22 samples, mean WS-23 content was 9.9mg/g (10.4 mg/g) in 7/22 samples, and mean WS-3 content was 0.4mg/g (0.2 mg/g) in 3/22 samples. In tested disposable devices, mean menthol content was in a similar range of 7.6mg/g (3.9mg/g) in 33/35 samples, but mean WS-23 content was much higher at 28.7mg/g (10.9mg/g) and present in all (35/35) samples, and mean WS-3 content was also increased at 2.8mg/g (1.3 mg/g) in 20/35 samples. Finally, for pod products, mean menthol content was 10.8mg/g (3.1mg/g) present in all 6/6 samples, but WS-23 was only present in one product at 2.3mg/g, and no WS-3 was detected (Table 1 & S2).

Comparing product flavor categorization to the results of “minty” compound presence, results indicate that 20/21 menthol-flavored products contained menthol, but 12/21 of these also contained other “minty” flavorants besides menthol (Table 2 & S2). One product labeled as menthol-flavored contained only WS-23 but no menthol. For mint/peppermint-flavored products, both distinctly-peppermint menthone and distinctly-spearmint L-carvone were highly prevalent (27/31 and 22/31, respectively) suggesting interchangeable use of the two mint varieties, or combinations thereof. This is supported by the finding that 6/6 spearmint-flavored products contained menthol, 6/6 contained menthone, and 5/6 contained L-carvone. While no wintergreen-flavored products were part of the identified top brands/flavors, mixed/concept-flavors contained a variety of “minty” flavorants (Table 2). The synthetic coolants WS-23 and WS-3 were present in all flavor categories, but their prevalence was notably high in the mint/peppermint (WS-23: 27/31 product, WS-3: 14/31) and mixed/concept categories (WS-23: 5/5, WS-3: 4/5; Table 2).

The 2023 data contains some repeat measurements of products purchased from different vendors, both in the disposable and the refill e-liquid categories (Table S2). Notably, these true duplicate measurements yielded very similar results in the refill e-liquid category which aligns with the reported high precision of the analytical technique and suggests high manufacturing reproducibility by the manufacturers. On the other hand, the one true replicate in the disposable category (Flum pebble menthol), did show similar ingredients, but a higher variability in the quantified components menthol, nicotine, WS-23, and WS-3 (Table S2).

### Nicotine content

Disposable and pod products of 2023 were labeled with nicotine content in mass/mass (as percentage) and thus experimentally determined nicotine contents in mass/mass (mg/g) could be compared to label information (conversion: %-value × 10 = value in mg/g): Deviations ranged from − 67% to + 15% (Table S2). After converting into absolute values, average nicotine deviation from label was 27% (standard deviation: 19%, N = 41). However, when separating the two categories it is apparent that this observation is driven by the disposable category, where the average nicotine deviation was 31% (18%, N = 35), while the average nicotine deviation for pod products was 4% (5%, N = 6). For 2019 samples and 2023 refill e-liquid samples, nicotine label information in mass/volume (mg/mL) could not be reliably compared to analytical quantification in mass/mass (mg/g); density measurements necessary for the conversion were not carried out. In addition, labels of 12 2023 products indicated synthetic nicotine use, 8 in the category of refill e-liquid, and 4 in the disposable category. Because the detection of synthetic nicotine is complicated due to the possibility of converting (synthetic) racemic nicotine into the (naturally occurring) (S)-enantiomer,^34^ these claims were not investigated further.

## Discussion

The results of this study suggest a partial substitution of menthol with synthetic cooling agents in the US e-liquid/e-cigarette marketplace between 2019 and 2023 as evidenced by the change in average menthol content (15.6 mg/g vs. 8.3mg/g, respectively). This result holds true even when the two e-liquids with menthol content > 180mg/g were excluded (11.2mg/g vs 8.3mg/g, respectively). Concurrently, while the synthetic coolants WS23 and WS-3 were only present in 1/104 (1%) and 11/104 (11%) samples in 2019, respectively, their prevalence in 2023 data increased to 43/63 (68%) and 23/63 (37%). This change seems to be largely driven by the introduction of disposable devices in the US marketplace in which the use of the synthetic coolant WS-23 at concentrations > 10 mg/g and up to > 50 mg/g seems to have become ubiquitous, as reported by several studies to date.^25–30^ Nonetheless, the prevalence of refill e-liquids containing synthetic coolants – especially WS-23 – also increased between 2019 and 2023 (WS-23: 1% vs. 32%; WS-3: 11% vs. 14%). The only category in 2023 with a low prevalence of synthetic cooling agent use are the pod-based systems, many of which have undergone the US FDA Premarket Tobacco Product Applications (PMTA) process, for which materials including e-liquid constituent information were submitted as early as 2020.^35^

In the 2019 data, two samples stand out, which contained 188mg/g and 217mg/g menthol (Table S1), which convert to 18.8% and 21.7% menthol by mass in the respective e-liquid. Such high concentrations of menthol are very likely aversive to most users, as menthol is known to also be an agonist of the irritant TRPA1 receptor.^36,37^ However, WS-23 and WS-3 activate TRPA1 to a lesser degree,^38^ while activating the cooling TRPM8 receptors, and in the case of WS-3 even more effectively than menthol.^31,39^ This raises the question if the observed partial replacement of menthol with the synthetic cooling agents WS-23 and WS-3 in the 2023 samples results in products that are less irritating at similar (or stronger) levels of cooling. Regulatory strategies that aim to restrict menthol in tobacco products due to its cooling effect should not be limited to the compound menthol alone, but should rather encompass all compound that elicits a cooling effect.^40^

While the use of WS-3 in ENDS products such as Juul has been reported by us as early as 2019,^24^ the finding of WS-23 in one 2019 sample (winter ice) is – to the best of our knowledge – the earliest report of WS-23 usage in ENDS/e-liquids.

The 2019 data showed a relatively high prevalence of the peppermint flavor compounds menthone, menthyl acetate, and eucalyptol, so it is reasonable to assume that peppermint oils, in which these compounds plus menthol can account for over 70% of flavorant content,^16^ were predominantly used to achieve peppermint flavor. In the 2023 data, menthyl acetate and eucalyptol prevalence decreased compared to 2019 samples (6% vs 78%, 45% vs. 65%, respectively; Table 2). In combination with the finding that peppermint/mint/spearmint samples in 2023 largely contained similar flavor profiles of menthol, menthone, eucalyptol, and L-carvone, it is likely that the use of peppermint oil was reduced in favor of using pure flavor compounds. In the context of findings of the carcinogen pulegone in mint oil, that may be preferable.^41,42^

Interestingly, while up to 59% of 2019 menthol-flavored samples also contained other “minty” flavorants, this number was reduced in the 2023 data to 38% of samples. Nonetheless, both datasets demonstrate that label information is not very reliable to distinguish between menthol, mint, peppermint, and spearmint as shown by chemical characterization (Table 2). As a result, it is likely hard for consumers to distinguish between these different flavor varieties and the use of a combined flavor category menthol/mint^13^ seems warranted, rather than separate ‘menthol’ and ‘mint’ categories. Importantly, these findings also suggest that careful analytical chemical analysis is important when studying effects of commercial tobacco products in the menthol/mint category.

The observed variation between labelled and quantified nicotine content in the disposable products (average deviation 31%; standard deviation: 18%, mostly lower than labeled) raises questions about the manufacturing process and reliability of label information of these often-imported products. In the same vein, a higher batch-to-batch variability in one disposable device (Flum Pebble ‘Menthol’; Table S2) was found, which was much less pronounced for duplicate analyses of refill e-liquids in the 2023 products (Table S2). Similar concerns about inconsistent batch-to-batch variability of disposable devices have also been suggested in a previous study.^43^

Several study limitations exist: For 2019 samples, no comprehensive strategy for identifying popular websites was established and it is possible that some were missed. Nonetheless, the relatively large sample size of 104 products should ensure a good representation of the market. For 2023 samples, popular e-cigarette/e-liquid websites were determined by unique visitor data, which does not necessarily translate into purchases on the sites. Nonetheless, the most frequented website had ~ 2.5x more unique visitors than the next two combined, suggesting its importance for the online market. In addition, the sample size of 64 samples was smaller than for 2019 samples, and divided into three product categories, which was a result of market changes after 2019. Further, we relied on website information on most popular products, which is information that could not be independently verified. Finally, analytical quantification was not carried out with triplicate injections; this was simply not feasible given the sample size and analysis run times of > 1h per sample. Nonetheless, we determined excellent precision of the analytical quantifications by %RSD values < 1%.

## Conclusions

This study presents average cooling agent contents – menthol, WS-23, and WS-3 – in US-marketed e-liquid and e-cigarette samples, as well as the presence of other “minty” compounds purchased in 2019 and 2023. Purchased samples reflect the change in the ENDS market between 2019 and 2023 toward a variety of products, such as refill e-liquids, disposables devices, and pod-based products. Results indicate an overall reduction in menthol content from 2019–2023, with a concurrent increase in synthetic cooling agent use. While only 1/104 samples in 2019 contained the cooling agent WS-23, its prevalence in 2023 was high (42/63 samples), and it was present in every disposable product tested (35/35) at an average concentration of 28.7mg/g. “Minty” flavor compound use was somewhat reduced in 2023 vs. 2019, and 2023 samples labeled as mint-, peppermint-, or spearmint-flavored all displayed similar flavorant profiles, warranting analytical verification if specific flavors are desired. Label information on nicotine was found accurate for pod-based products, but a high average label deviation of > 30% was found for disposable devices, raising questions around manufacturing practices of these devices.

## Supplementary Material

Supplementary Files

This is a list of supplementary files associated with this preprint. Click to download.

- 2025125MentholmintSIV3.pdf

## Figures and Tables

**Table 1:** Overview of quantified coolants: Menthol (both 2019 & 2023), WS-23, and WS-3 (both 2023 only). Data is presented as mean (stdev) in mg/g; N – number of products; minimum (min.) and maximum (max.) content found. Abbreviations: n.d. – not detected; n.q. – not quantified.

	2019 data (mg/g)	2023 data (mg/g)
**Overall coolant content**		
	**All samples** **(N = 104)**	**All samples** **(N = 63)**
Menthol	15.6 (31.0); N = 87 Min.: 0.4; Max.: 217.3	8.3 (4.1); N = 60 Min.: 0.2; Max.: 18.4
WS-23	N = 1^a^ 2.7^b^	25.0 (13.2); N = 43 Min.: 1.7; Max.: 54.6
WS-3	N = 11^a^ 2.0 (3.3)^b^, N = 9^b^ Min.: 0.2^b^; Max.: 10.3^b^	2.5 (1.5); N = 23 Min.: 0.2; Max.: 5.1
**Coolant content by device type**		
	Refill e-liquid (N = 104)	Refill e-liquid (N = 22)
Menthol	15.6 (31.0); N = 87	8.8 (4.5); N = 21
WS-23	n.q.; N = 1	9.9 (10.4); N = 7
WS-3	n.q.; N = 11	0.4 (0.2); N = 3
**Coolant content by device type**	**Disposable devices** **(N = 0)**	**Disposable devices** **(N = 35)**
Menthol		7.6 (3.9); N = 33
WS-23		28.7 (10.9); N = 35
WS-3		2.8 (1.3); N = 20
**Coolant content by device type**	**Pod devices** **(N = 0)**	**Pod devices** **(N = 6)**
Menthol		10.8 (3.1); N = 6
WS-23		2.3; N = 1
WS-3		n.d.

a 2019 qualitative data

b 2025 re-quantification results of 2019 samples with positive, qualitative coolant identification. Mean, standard deviation, min., and max. values based on 1 sample containing WS-23, and 9 samples containing WS-3 (2 samples with WS-3 concentration < LOQ; see Table S1).

**Table 2:** Overview of “minty” flavor compound and synthetic cooling agent presence by flavor category and testing year. Data is presented as # of samples containing the listed compound (per column) in %, and mean (stdev) in mg/g if quantified (Menthol 2019 & 2023; WS-23 and WS-3 only in 2023). Green highlights indicate expected compounds based on flavor description.

2019 data								
Flavor \ compound	Menthol	WS-23	WS-3	Menthone	Menthyl acetate	Eucalyptol	L-Carvone	Methyl salicylate
**Menthol (N=29)**	29 (100%) 24.9 (39.0)	0 (0%)	2 (7%)	17 (59%)	13 (45%)	9 (31%)	5 (17%)	3 (10%)
**Mint/peppermint (N=23)**	22 (96%) 6.3 (5.0)	0 (0%)	1 (4%)	19 (83%)	18 (78%)	15 (65%)	9 (39%)	1 (4%)
**Spearmint (N=15)**	10 (67%) 3.6 (3.4)	0 (0%)	2 (13%)	10 (67%)	8 (53%)	8 (53%)	15 (100%)	1 (7%)
**Wintergreen (N=10)**	1 (10%) 1.0	0 (0%)	0 (0%)	1 (10%)	0 (0%)	0 (0%)	0 (0%)	9 (90%)
**Mixed/Concept (N=27)**	25 (93%) 18.4 (37.2)	1 (4%)	6 (22%)	21 (78%)	17 (63%)	19 (70%)	10 (37%)	11 (41%)
**Total (N=104)**	**87 (84%)** **15.6 (31.0)**	**1 (1%)**	**11 (11%)**	**68 (65%)**	**57 (55%)**	**51 (49%)**	**39 (38%)**	**25 (24%)**
2023 data								
Flavor \ compound	Menthol	WS-23	WS-3	Mentone	Menthyl acetate	Eucalyptol	l-Carvone	Methyl salicylate
**Menthol (N=21)**	20 (95%) 10.0 (3.7)	7 (33%) 19.2 (15.6)	5 (24%) 1.8 (1.5)	8 (38%)	2 (10%)	4 (19%)	7 (33%)	3 (14%)
**Mint/peppermint (N=31)**	31 (100%) 7.5 (4.0)	27 (87%) 23.7 (11.6)	14 (45%) 2.8 (1.4)	27 (87%)	2 (6%)	14 (45%)	22 (71%)	1 (3%)
**Spearmint (N=6)**	6 (100%) 9.5 (3.3)	4 (67%) 23.9 (12.1)	0 (0%)	6 (100%)	0 (0%)	4 (67%)	5 (83%)	0 (0%)
**Wintergreen (N=0)**								
**Mixed/Concept (N=5)**	3 (60%) 3.6 (4.4)	5 (100%) 41.3 (8.9)	4 (80%) 2.3 (1.9)	2 (40%)	0 (0%)	1 (20%)	2 (40%)	0 (0%)
**Total (N=63)**	**60 (95%)** **8.3 (4.1)**	**43 (68%)** **25.0 (13.2)**	**23 (37%)** **2.5 (1.5)**	**43 (68%)**	**4 (6%)**	**23 (37%)**	**36 (57%)**	**4 (6%)**
